# The *Tc1/mariner* transposable element family shapes genetic variation and gene expression in the protist *Trichomonas vaginalis*

**DOI:** 10.1186/1759-8753-5-12

**Published:** 2014-04-24

**Authors:** Martina Bradic, Sally D Warring, Vivien Low, Jane M Carlton

**Affiliations:** 1 Center for Genomics and Systems Biology, Department of Biology, New York University, 12 Waverly Place, New York, NY 10003, USA

**Keywords:** DNA transposable element, *Mariner* transposase, *Trichomonas vaginalis*, Population genetics, Gene expression

## Abstract

**Background:**

*Trichomonas vaginalis* is the most prevalent non-viral sexually transmitted parasite. Although the protist is presumed to reproduce asexually, 60% of its haploid genome contains transposable elements (TEs), known contributors to genome variability. The availability of a draft genome sequence and our collection of >200 global isolates of *T. vaginalis* facilitate the study and analysis of TE population dynamics and their contribution to genomic variability in this protist.

**Results:**

We present here a pilot study of a subset of class II *Tc1/mariner* TEs that belong to the *T. vaginalis Tvmar1* family. We report the genetic structure of 19 *Tvmar1* loci, their ability to encode a full-length transposase protein, and their insertion frequencies in 94 global isolates from seven regions of the world. While most of the *Tvmar1* elements studied exhibited low insertion frequencies, two of the 19 loci (locus 1 and locus 9) show high insertion frequencies of 1.00 and 0.96, respectively. The genetic structuring of the global populations identified by principal component analysis (PCA) of the *Tvmar1* loci is in general agreement with published data based on genotyping, showing that *Tvmar1* polymorphisms are a robust indicator of *T. vaginalis* genetic history. Analysis of expression of 22 genes flanking 13 *Tvmar1* loci indicated significantly altered expression of six of the genes next to five *Tvmar1* insertions, suggesting that the insertions have functional implications for *T. vaginalis* gene expression.

**Conclusions:**

Our study is the first in *T. vaginalis* to describe *Tvmar1* population dynamics and its contribution to genetic variability of the parasite. We show that a majority of our studied *Tvmar1* insertion loci exist at very low frequencies in the global population, and insertions are variable between geographical isolates. In addition, we observe that low frequency insertion is related to reduced or abolished expression of flanking genes. While low insertion frequencies might be expected, we identified two *Tvmar1* insertion loci that are fixed across global populations. This observation indicates that *Tvmar1* insertion may have differing impacts and fitness costs in the host genome and may play varying roles in the adaptive evolution of *T. vaginalis*.

## Background

Transposable elements (TEs) are mobile genetic units that exhibit broad diversity in their structure and transposition mechanisms. They are present in many eukaryotic genomes and their movement and accumulation represent a major force shaping the genes and genomes of almost all organisms. TEs are typically divided into two classes: class I that transposes via an RNA intermediate; and class II that transposes via a DNA intermediate. There are three major subclasses of class II DNA transposons: 1) those that excise as double-stranded DNA and reinsert elsewhere in the genome, that is, the classic ‘cut-and-paste’ transposons [1]; 2) those that utilize a mechanism probably related to rolling-circle replication, such as *Helitrons *[2,3]; and 3) *Mavericks*, whose mechanism of transposition is not yet well understood, but that likely replicate using a self-encoded DNA polymerase [4]. Special characteristics of cut-and-paste transposons include a central protein coding region that encodes a transposase that is required for transposition flanked by (with a few exceptions) terminal inverted repeats (TIRs).

While the contribution of TEs to the genomes of many higher eukaryotes has been well described, we know little about TE presence and influence in the genomes of single-celled eukaryotes. Parasitic protists have compact genomes (approximately 10 to 80 Mb [5]), and contain from none (for example, the malaria parasite *Plasmodium falciparum*) to >14% (for example, *Entamoeba histolytica*) TE repeats that are correlated with their haploid genome sizes [6,7]. However, unlike other parasites of human health importance, the sexually transmitted parasite *Trichomonas vaginalis* has a large genome of approximately 160 Mb, two thirds of which consists of TE repeats, predominantly class II DNA transposons [1,4,8,9]. Recent studies indicate that the large genome size of *T. vaginalis* can be largely accounted for by the massive amplification of *Maverick* TEs [4] that are present in approximately 3,000 copies in the genome. The average size of *Maverick* elements in *T. vaginalis* is 15 to 20 Kb, thus they probably occupy approximately 60 Mb (37%) of the 160 Mb genome. Their likely significant impact on genome dynamics has been hypothesized [4].

In addition to *Mavericks*, a family of DNA transposons belonging to the ubiquitous *Tc1/mariner* superfamily, *Tvmar1*, is present in the *T. vaginalis* genome in over 1,000 copies. *Tvmar1* was the first representative of a *mariner* family member to be found in a protist, and is one of only a handful of active *mariner* elements found in any species [10]. The *Tvmar1* family is highly specific to *T. vaginalis* since very closely related homologs could not be detected by Southern blot in other species of trichomonad such as *Trichomonas tenax* and *Pentatrichomonas hominis *[10], suggesting recent acquisition in the *T. vaginalis* lineage. Thus the *Tvmar1* family may play an important role in *T. vaginalis* speciation and adaptation [10].

The large size of the *T. vaginalis* genome is thought to be due to the high copy number of TE families [1,8,9]. TE abundance is correlated with genome size, which is further correlated with cell size across different phyla [11-14]. Cell size is an important factor for *T. vaginalis* parasitism as the larger the cell, the more surface with which *T. vaginalis* has to adhere to host epithelium cells, a crucial factor in its pathogenicity. *Tritrichomonas foetus*, which parasitizes the urogenital tract of cattle, is another trichomonad with a similarly large genome (approximately 177 Mb), which may be caused by TE load [15]. Although yet to be tested, it is tempting to speculate that expansion of TE families could be the raw material that provides the variation upon which natural selection could act, favoring the largest cells [8]. To what extent TEs shape genetic diversity among *T. vaginalis* isolates and whether the benefits of a large genome size are enough to counteract the potentially deleterious effects of TE insertions in or near host genes is an important question. In this study, we aimed to move closer to answering these points by investigating the abundance and distribution of a subset of 19 *Tvmar1* loci in 94 global isolates of *T. vaginalis*. In addition we sought to determine the effect of *Tvmar1* insertions on host gene expression and the functional implications of such insertions.

## Results

### Characterization of *Tvmar1* elements in the *T. vaginalis* genome

Approximately 1,000 *Tvmar1* elements are currently annotated in the G3 reference genome, although many appear fragmented due to an incomplete assembly caused by the repetitive nature of the *T. vaginalis* genome. To identify complete *Tvmar1* elements (defined as those that contain no ambiguous base calls and are flanked by 3′ and 5′ TIRs [16]) for use in this study, we screened the reference genome in TrichDB [17]. A total of 408 intact elements were identified and their DNA sequences aligned for characterization (data not shown). The sequences were found to be highly similar, with an average pairwise difference of 0.006 and a mode length identical to the consensus sequence of 1,304 bp. We classified the 408 elements as putatively autonomous (those that retain the ability to encode a transposase protein identical in amino acid sequence to the consensus) or nonautonomous (derivatives of autonomous elements that have acquired disruptive mutations in the transposase open reading frame (ORF), such that they can still be activated in the presence of a transposase transcribed from an autonomous element). Of the 408 elements, 33 were classified as autonomous. The remaining 375 contained at least one nonsynonymous substitution within the transposase ORF (184 elements), or an indel or nonsense mutation that truncated the transposase ORF (191 elements). It is worth noting that these nonsynonymous substitutions may not interfere with the function of the transposase protein, and thus the number of autonomous elements may be higher.

### *Tvmar1* exhibits insertion polymorphism

In order to test our hypothesis that *Tvmar1* TEs exhibit insertion polymorphism, we designed a PCR assay that would identify the presence or absence of *Tvmar1* insertions in the genomes of a wide variety of *T. vaginalis* isolates (Methods). The *T. vaginalis* reference genome assembly was screened and unique oligonucleotide primers identified that flanked a subset of 19 full-length *Tvmar1* elements, approximately 5% of the total full-length elements found in the reference genome sequence. The elements were selected at random and the only criterion for their selection was the presence of sufficient unique flanking sequence in order that we could design element-specific primers. The SNP calls and indels of the 19 *Tvmar1* elements were confirmed by Sanger sequencing. A file showing the SNP calls and indels is shown (see Additional files 1 and 2). Of the 19 elements, eight are putatively nonautonomous, with three containing indels that truncate the transposase ORF, and five elements containing nonsynonymous mutations within the transposase. The remaining eleven elements encode a transposase protein identical to the consensus sequence and are putatively autonomous. Each of the elements was assigned a locus number, and this subset of elements is referred to in the remainder of this study. Details of the 19 loci used in the study are provided (see Additional file 3).

**Figure 1:**
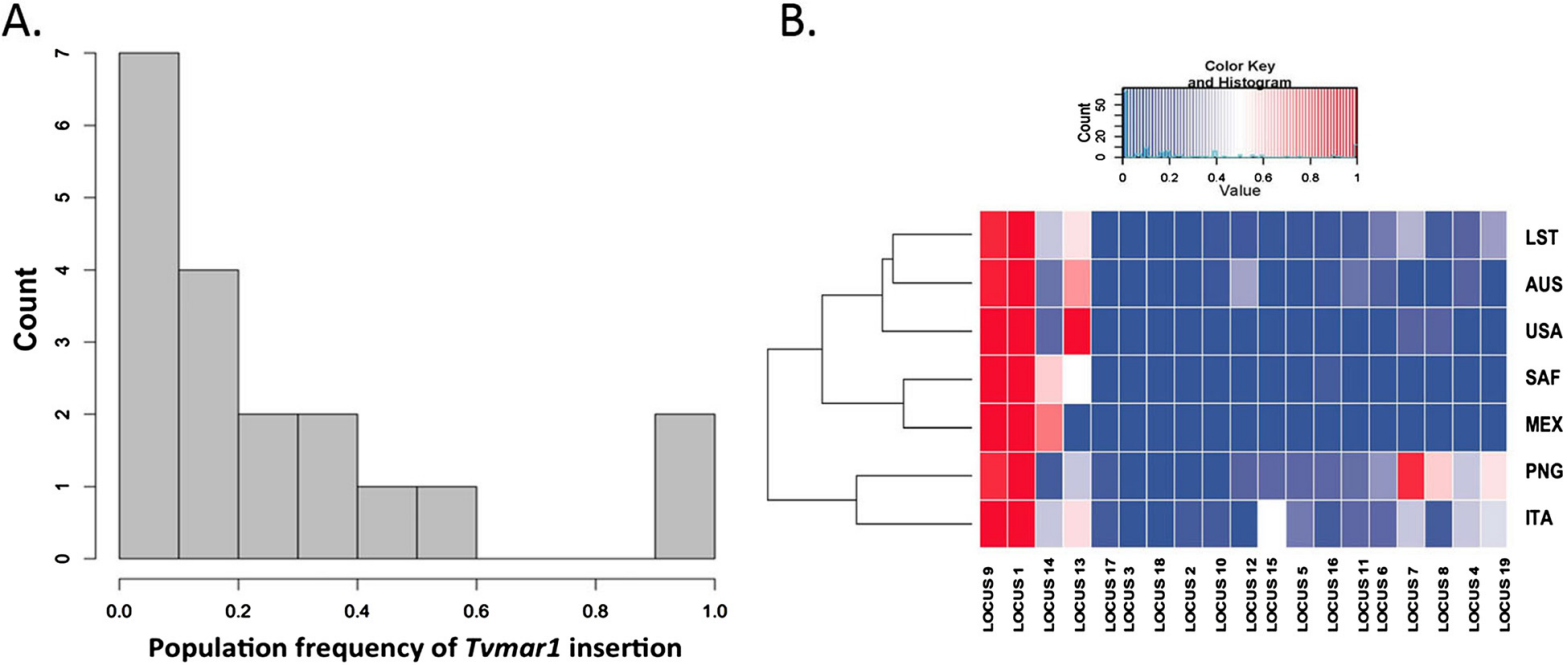
**Tvmar1 allele frequency spectrum and insertion frequencies. (A)***Tvmar1* allele frequency spectrum in a panel of 94 *T. vaginalis* isolates. The x-axis represents frequencies, while the y-axis represents counts. **(B)***Tvmar1* insertion frequencies in a panel of 94 *T. vaginalis* isolates. The gradient of *Tvmar1* frequencies (from 0.0 to 1.0) identified in different isolates is represented as a heatmap, with colors indicating the following: blue represents no insertion; white represents intermediate frequencies; and red represents fixation or elements that are close to fixation. Two dendrograms are shown. The top dendrogram represents the clustering of loci based on their similarity by frequency. The dendrogram on the left represents clustering based on the frequency of all the loci used in the study. AUS, Australia; ITA, Italy; LST, laboratory strains; MEX, Mexico; PNG, Papua New Guinea; SAF, South Africa; USA, United Sates of America.

We performed our PCR assay on 94 global *T. vaginalis* isolates from: the United States of America (USA, total 41), Mexico (MEX, total 10), Italy (ITA, total 5), South Africa (SAF, total 10), Australia (AUS, total 7) and Papua New Guinea (PNG, total 13), and several commonly used laboratory strains (LST, total 8). A file describing the isolates used in this study is provided (see Additional file 4). For each isolate, a PCR reaction using the unique flanking primers was performed, followed by a second PCR reaction with an internal transposon primer to confirm the result. A table of insertion genotypes by locus and isolate is shown (see Additional file 5). Only known *Tvmar1* insertions identified in the G3 reference strain were screened for in other strains. From a total of 1,877 PCRs performed in duplicates, 376 insertions were found, with an overall PCR success rate of 87.2% (lowest success rate for any locus, 67.4%; highest success rate for any locus, 98.9%). As anticipated, due to the potentially deleterious nature of transposon insertions in gene-rich regions, most *Tvmar1* elements exhibited a low level of insertion in a majority of the isolates (Figure 1A). In 14 out of 19 loci, less than 25% of isolates had an insertion, and in 16 out of 19 loci, less than 50% of isolates had an insertion. Locus 18 was found to be present in the reference G3 strain only and no other isolates.

In particular, loci 1, 9 and 13 showed a high degree of insertion, with locus 1 and locus 9 being fixed or close to fixation in all isolates (Figure 1B). A second group consisting of loci 4, 7, 8 and 14 showed intermediate frequencies in some of the populations. A third group consisting of loci 2, 3, 10, 17 and 18 showed no insertions in most of the populations. The total insertion frequency in the SAF population was the highest (36.1%), followed by the USA population with 25.9%.

### Population structure and differentiation of *T. vaginalis* isolates revealed by *Tvmar1* as a genetic marker

Understanding the population dynamics of the *Tvmar1* elements in *T. vaginalis* will provide important clues as to the evolutionary history of the family. Towards this goal, we next compared the population genetic structuring of *T. vaginalis* determined using *Tvmar1* as a genetic marker with the results of our previous population genetic analyses using a panel of validated microsatellite and SNP markers and a similar set of global isolates [18,19]. Briefly, these previous studies revealed high genetic diversity of *T. vaginalis*, and two genetic ‘types’ or lineages that show an unusual population structure in that they are distributed in near-equal frequencies worldwide [19].

**Figure 2:**
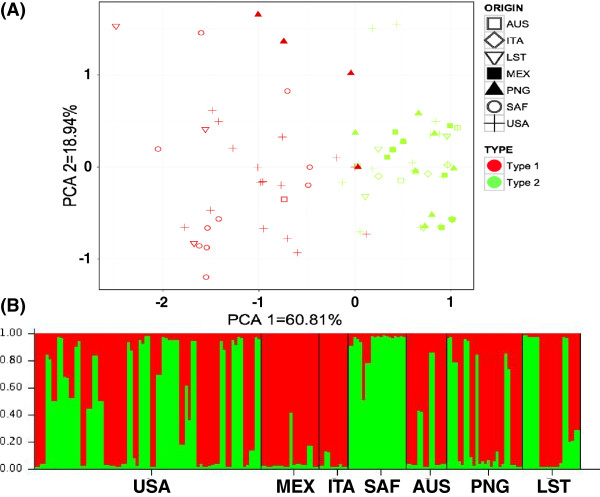
***T. vaginalis *population structure based on 19 *Tvmar1 *insertions. (A)** Principal component analysis (PCA) using seven geographical groups. A shape represents each isolate and color represents genetic type 1 and 2. The first three PCA axes explain the highest percentage of the variation (60.81% first, 18.94% second and 10.53% third). **(B)** Structure analysis of *T. vaginalis* isolates using *K* = 2, determined as the optimal *K* number. Each individual is represented by a thin vertical line, which is partitioned into *K* segments that represent its estimated population group membership fractions. Black lines separate groups of isolates. AUS, Australia; ITA, Italy; LST, laboratory strains; MEX, Mexico; PCA, principal component analysis; PNG, Papua New Guinea; SAF, South Africa; USA, United Sates of America.

Similar to our previous findings, no geographical population differentiation of *T. vaginalis* isolates was found using *Tvmar1* as a genetic marker. However, principal component analysis (PCA) of the *Tvmar1* insertion frequencies and use of the Bayesian clustering program STRUCTURE [20] to determine the number of optimal *K* populations according to *Tvmar1* frequencies at each locus, identified the two distinct *T. vaginalis* lineages (type 1 and type 2) identified by previous studies (Figure 2). We tested significance of this two-type population structuring by using hierarchical analysis of molecular variance (AMOVA), and found that applying no groupings to the populations was found to be significant (*P* <0.000001), and grouping populations based on their genetic type as suggested by the PCA results and contrasting the SAF group versus all other groups was also significant (*P* <0.000001). Details of AMOVA structuring and significance test are shown (see Additional file 6). Overall, these results show that there is evidence of two genetically distinct groups, as previously identified [19]. *Tvmar1* elements reflect the genetic history of their host genome, similar to standard genetic markers, and support the existence of the two *T. vaginalis* genetic types identified previously.

### *Tvmar1* insertion is associated with changes in host gene expression

Next we undertook experiments to determine the effect of *Tvmar1* insertions on the expression of nearby *T. vaginalis* genes. Quantitative reverse transcription PCR (qRT-PCR) was used to quantitate the mRNA expression of a total of 22 genes flanking 13 of the 19 *Tvmar1* insertion sites. A total of 34 genes that could be identified flanking the 19 *Tvmar1* loci in the genome annotation were initially analyzed, but genes flanking six loci (loci 1, 2, 7, 9, 14 and 18) had to be discarded for various reasons including lack of primer specificity. From ten to 15 isolates consisting of insertion positive and insertion negatives for the 13 *Tvmar1* loci were analyzed in duplicate for expression of each of the genes flanking the loci, a total of 624 qRT-PCR reactions. A list of primers used to determine the expression of *Tvmar1* flanking genes is provided (see Additional file 7).

**Figure 3:**
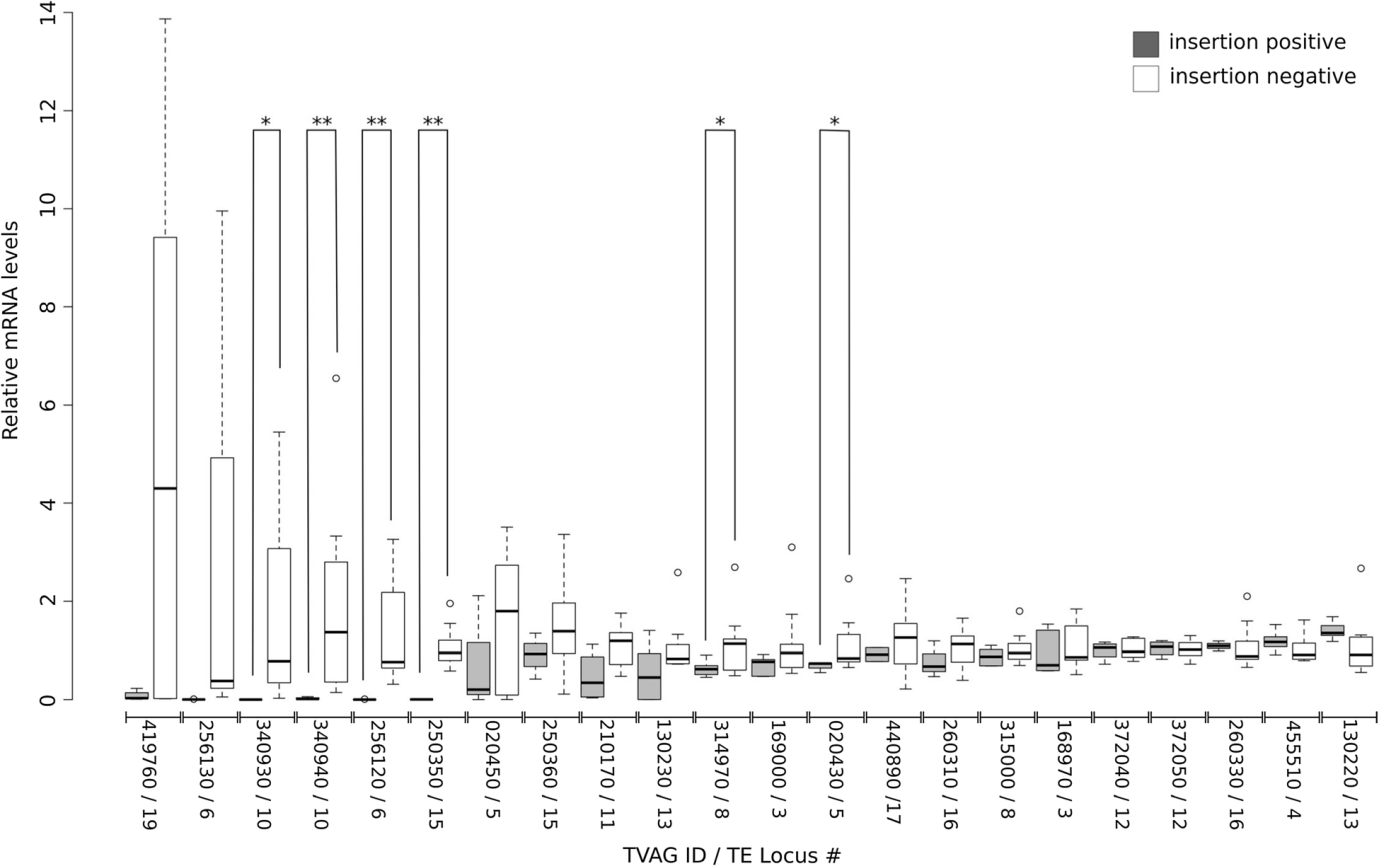
**mRNA levels of genes flanking *Tvmar1 *loci in insertion positive and insertion negative isolates.** Box plot showing relative mRNA levels for 22 genes flanking 13 *Tvmar1* loci from insertion positive (grey, left side) and insertion negative (white, right side) isolates. The genes have been ordered by decreasing mean difference in mRNA levels between insertion positive and insertion negative groups. A single asterisk above a box plot pair indicates a *P* value of <0.05, while a double asterisk indicates a *P* value <0.01 (Welch Two Sample *t*-test). The gene examined and its associated *Tvmar1* locus is indicated on the x-axis (the tag ‘TVAG’ has been removed due to space constraints). Five outlier data points have been excluded from the insertion positive groups of TVAG_020450, TVAG_256130, TVAG_340930 and TVAG_419760, to allow for higher resolution on the y-axis; however, these outliers are included in the statistical analyses.

In the majority of cases, gene expression was reduced in insertion positive isolates, that is, those parasites that contained a *Tvmar1* element close to the gene analyzed (Figure 3). In particular, six genes (TVAG_020430, TVAG_256120, TVAG_314970, TVAG_340930, TVAG_340940 and TVAG_250350) flanking five *Tvmar1* loci (loci 5, 6, 8, 10 and 15) showed significant reduction or abolition of relative mRNA levels in insertion positive isolates compared with insertion negative isolates. The differences between the insertion positive and insertion negative groups were not significant for 12 genes, and interestingly four genes (TVAG_372050, TVAG_260330, TVAG_455510 and TVAG_130220) showed increased expression in insertion positive isolates, although the differences were also not significant (Figure 3). Thus, insertion of a *Tvmar1* element close to a gene appears to affect that gene’s transcription.

**Figure 4:**
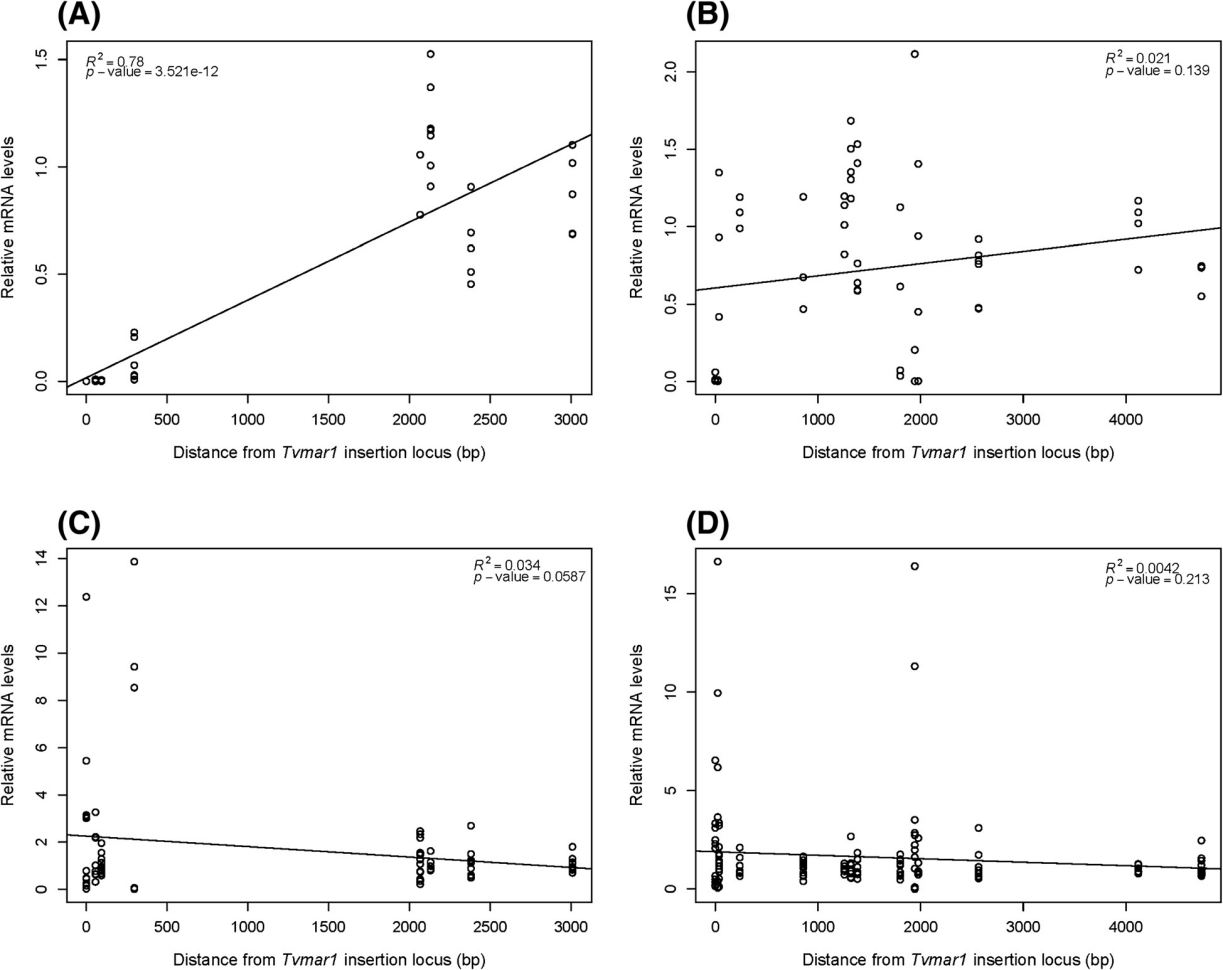
**Distance and orientation plots for *Tvmar1 *insertions and gene expression.** Linear regression plots of relative mRNA levels of 22 genes in 15 isolates plotted against distance in base pairs (bp) between a *Tvmar1* insertion site and its nearest gene. The plots are divided into relative mRNA levels of proximal genes with *Tvmar1* insertions located: **(A)** 5′ and **(B)** 3′ to the gene in insertion positive isolates; and **(C)** 5′ and **(D)** 3′ to the gene in insertion negative isolates.

To examine if this apparent effect of *Tvmar1* insertion on *T. vaginalis* gene expression correlates with distance from the insertion site and whether the insertion is 5′ or 3′ to the gene, we plotted relative mRNA levels with distance from the *Tvmar1* locus for insertion positive isolates (Figure 4). We found that mRNA expression is positively correlated with increasing distance from the *Tvmar1* locus for genes that have a *Tvmar1* insertion located 5′ to them (Figure 4A, B), an effect that is not seen in the absence of a *Tvmar1* element (Figure 4C, D).

## Discussion

### *Tvmar1* insertion variation in the genome of *T. vaginalis*

Despite the major contribution that TEs make to the genome size and gene complement of *T. vaginalis*, little is known about how these sequences have shaped the evolution of the parasite’s genome. Indeed, relationships between TE load and parasitic life-cycle are largely understudied [21]. Intracellular parasites usually have a low TE load or no TEs at all, perhaps due to the evolutionary pressure to maintain a small cell size and thus a small genome [22,23]. For example, pathogenicity of the extracellular parasite *Entamoeba histolytica* has been correlated with genome differences caused by TE insertions, when comparing pathogenic *E. histolytica* with non-pathogenic *Entamoeba dispar *[24]. *T. vaginalis* is an extracellular parasite for which it has been hypothesized a large cell size may be highly beneficial [1,8,9]. Here, we sought to understand the contribution that one major group of TEs, the *T. vaginalis Tc1/mariner* family *Tvmar1*, might play in shaping the evolution of this genome and the functional consequences this may have for parasite fitness.

Our initial studies looked at the presence of 19 *Tvmar1* loci in 94 global *T. vaginalis* isolates. A high level of polymorphism in the point of insertion was identified, with most loci showing low insertion frequencies in isolates from all geographic regions. Only two loci showed a high insertion rate: the first insertion (locus 1) was present in all 94 global isolates studied, that is, it was fixed; and the second (locus 9) had an insertion frequency of 96.2%, compared to an average insertion frequency of 24.7% for the other 17 loci in all isolates. This unusually high rate of insertion for two loci may be explained by two possible hypotheses. First, the insertions could provide potential genetic novelty and potential adaptive value for the parasite, as has been demonstrated for TE insertions in other studies [25-28]. Second, these insertions may have a neutral effect on the surrounding genome, and thus have reached fixation due to genetic drift. These loci present intriguing candidates for further investigation to determine why we see close to fixation/fixed TE insertions in *T. vaginalis*. In addition, the presence of so many polymorphic insertions in our data provides strong support for previous suggestions that *Tvmar1* is still an active *mariner* element and recently amplified in the *T. vaginalis* genome [10].

Using the *Tvmar1* insertions as a form of genetic marker, we found that the diversity of insertions was high for most of the loci and partitioned within and between geographical regions. The genetic groups (type 1 and type 2) identified using PCA and STRUCTURE clustering were in agreement (with some exceptions that did not represent the same genetic type) with published data based on SNPs and microsatellites [19]. A table of comparative parasite genetic types as determined by microsatellite and TE polymorphism data is shown (Additional file 8). This indicates that *Tvmar1* polymorphisms reflect the genetic history of *T. vaginalis*. It was also apparent that the SAF (South African) population may differ more in its origin from other populations and that it may represent an older, distinct lineage as suggested in our previous work [19]. However, these SAF isolates were collected from just two clinics, which may have introduced sample bias in this collection of isolates.

### *Tvmar1* affects host gene expression

We provide the first evidence that TEs of the *mariner* family affect gene expression in *T. vaginalis*. We found six genes that exhibited significantly reduced or abolished mRNA levels in the presence of a proximal *Tvmar1* insertion, and furthermore, we found that gene expression is positively correlated with increasing distance from *Tvmar1* elements. As the annotations of these six genes give little information as to their function, it is hard to predict the consequences of altered gene expression on the parasites. These pilot data pave the way for a larger genome-wide analysis of the effect of *Tvmar1* on the *T. vaginalis* genome.

TEs can interrupt gene expression by inserting directly into the coding region of a gene, or into a gene’s regulatory sequences, or by inducing epigenetic silencing of nearby genes [29,30]. Our results provide clues as to the mechanism of decreased gene expression for certain genes flanking *Tvmar1* elements. First, the ORF of one gene (TVAG_340940) is interrupted by a *Tvmar1* insertion at its 3′ and shows abolished expression in insertion positive isolates, suggesting that, as might be expected, *Tvmar1* elements can disrupt gene expression by inserting directly into coding regions. Second, we found that the correlation between distance from a *Tvmar1* insertion and gene expression was only significant when the insertion is located 5′ to the gene. In addition, significant changes in gene expression were observed in genes located up to 4.7 Kb (for example, TVAG_020430) from the nearest *Tvmar1* element, while many genes that do not show significant changes were located relatively close (for example, within 36 bp for TVAG_250360). These findings suggest that *Tvmar1* insertions can also affect the expression of nearby genes, and that it is not the proximity to the element that is the major determinate of gene downregulation, but rather the interruption of specific cis-regulatory sequence (s).

The effect of *Tvmar1* insertions on transcription shown here suggests that some *Tvmar1* insertions may be selected against and actively purged from the genome due to their influence on gene function and their effect on host fitness. However, it is important to note that there is likely very little ‘neutral space’ in the 177 Mb *T. vaginalis* assembly, which contains a predicted set of approximately 60,000 protein coding genes of average size 929 bp and gene density of 2,956 bp (that is, one gene is found every 3 Kbp) [8]. If there is little ‘neutral’ space in the genome for *Tvmar1* insertions to land, then many of them may have deleterious effects, because they will invariably disrupt genes or their regulatory sequences. Moreover, the set of insertions that we analyzed here is biased towards gene-damaging insertions, because unique flanking sequences were required for PCR amplification, and *Tvmar1* insertions found in non-unique regions of the genome could not be assayed with these methods.

In addition to purifying selection that purges *Tvmar1* insertions from the *T. vaginalis* genome, other host mechanisms or self-regulation could regulate *Tvmar1* load and activity in this unicellular eukaryote. So far none of the host defense mechanisms against TEs (methylation, chromatin modification, RNAi pathways or DNA editing) known to regulate TE insertion in other organisms [31,32] have been described as functional in *T. vaginalis *[33]. The presence of RNAi homologs in the genome [1,8,9] and miRNA candidates [34,35] suggests that *T. vaginalis* may have an RNAi pathway and thus a potential regulatory mechanism for TE insertion [36]. More studies are needed to explore the roles of these epigenetic mechanisms in *T. vaginalis Tvmar1* dynamics and their functional implications for host gene expression.

Clearly, *mariner* element transposition has functional implications for *T. vaginalis* and provides an important source of genetic variation on which natural selection can act and produce adaptation.

However, other TE families should be examined to elucidate whether they play a similar important role in shaping *T. vaginalis* genome evolution. An important question is whether the fate of *T. vaginalis* is doomed due to its extreme TE load and a presumed asexual lifestyle, or it has sufficient mechanisms to purge deleterious TE insertions and thus create a balance between TE gain and loss, allowing for the evolution of novelty and adaptation. Our future goals will be to understand which conditions promote *Tvmar1* mobilization, and how we can harness this extraordinary burden as a tool for gene transfection and silencing, and ultimately to control *T. vaginalis* infections.

## Conclusions

Our study is the first to provide evidence of *mariner* element dynamics and the contribution of this transposable element family to the genetic variability of the protist *T. vaginalis*. Here we have shown that *Tvmar1* insertion sites are polymorphic in a set of 94 global *T. vaginalis* isolates, and that the majority of insertions are present at low frequency. While the finding of low *Tvmar1* insertion frequency is not unexpected due to the known deleterious effect of TEs, we nevertheless identified two fixed *Tvmar1* loci in the 94 isolates. In addition, we observed that in several instances low frequency insertions are related to either reduced or abolished mRNA expression of nearby *T. vaginalis* genes. This observation indicates the potential impact of *Tvmar1* on the adaptive evolution of *T. vaginalis*, and underlines the importance of further work on the TE burden of the parasite.

## Methods

### Source of *T. vaginalis* isolates and DNA

The 19 *Tvmar1* elements used in this study were previously identified [10] and are annotated in the current annotation of the *T. vaginalis* G3 strain genome build 1.3 represented in TrichDB (http://www.trichdb.org). A total of 94 *T. vaginalis* isolates from six different regions of the world and characterized as previously described [19] were used (Additional file 4). All isolates were cultured in modified Diamond’s media with the supplement of 10% horse serum, penicillin and streptomycin (Invitrogen, Carlsbad, CA, USA), and iron solution composed of ferrous ammonium sulfate and sulfosalicylic acid (Thermo Fisher Scientific, Waltham, MA, USA) [19]. We used a standard DNA phenol-chloroform method for DNA extraction [19].

### PCR primer design and *Tvmar1* element amplification

To find *Tvmar1* insertions in the global set of 94 isolates, a PCR-based assay was designed such that amplification of a unique sized band would unambiguously indicate the presence or absence of an element. Briefly, the sequence of *Tvmar1* [GenBank:AY282463] was entered into the TrichDB BLAST function, and full-length matches selected. Unique PCR primer pairs were designed using oligonucleotide calculator software (Oligo Calc: http://www.basic.northwestern.edu/biotools/oligocalc.html) to the flanking regions either side of the *Tvmar1* locus in the reference genome (Additional file 3) A third and fourth primer were designed to internal sequences of the transposase, and these were used with a flanking primer to confirm an insertion. Primer Int1 (5′-AAACTTCTTGGATTGATACGCACCC-3′) was used with the forward flanking primer and Int2 (5′-TGTCGGTTTTTTGGGGCGTGAATG-3′) with the reverse flanking primer, because amplification was improved in some instances using these different combinations. PCRs were performed in 96-well plates and the reference strain G3 was used as a control in each plate to confirm the locus and amplification efficiency of each reaction. PCR was performed in 25 μl reaction volumes using approximately 25 ng of template DNA, 0.5 μl of each primer (10 pmol/μl) and GoTaq® Green Master Mix (Promega, Madison, WI, USA) in the following conditions: 2 minutes at 95°C (1 minute at 95°C, 2 minutes at the annealing temperature and 4 minutes at 72°C) × 30, and 5 minutes at 72°C. PCR products were sized by electrophoresis on a 1% agarose gel with EtBr in a 1 × TAE buffer and visualized under UV light. Sanger sequencing (two-fold coverage) of all PCR products confirmed the presence or absence of *Tvmar1* elements. Clustal Omega alignment [37] of the sequences was used to identify if the element retained autonomous element characteristics (an intact transposase gene) or if it was non-autonomous (with deletions and mutations in the transposase gene).

### Population frequencies and structure analysis

Frequencies of insertions were calculated using PCR data for each isolate and customized scripting in R. The heatmap of frequency data for geographical origin was designed using the *heatmap.2* function in the R gplots package (http://CRAN.R-project.org/package=gplots). Population structure was examined using a non-model-based multivariate analysis method as implemented in the adegenet 1.2-2 package using the *dudi.pca* function [38]. In addition, we used the Bayesian clustering program STRUCTURE 2.2 to determine the number of optimal *K* populations according to *Tvmar1* frequencies at each locus [20]. The program was run repeatedly ten times for each of the *K* values (*K* = 1–7) with 1 × 10^6^ iterations followed by 1 × 10^5^ Markov chain Monte Carlo (MCMC) repeats. The optimal *K* was determined by data probabilities calculation (Pr (X | *K*)) and Δ*K*, the rate of change in the log probability of data between successive *K* values, was determined using Evanno method and Structure harvester [39,40].

We evaluated the relative contribution between groups, within groups and within populations using AMOVA [41], performed using Arlequin version 3.0 [42] for 20,000 permutations. We tested the null hypothesis of panmixia (all populations together) as well as structuring revealed by PCA, using South Africa isolates as a group versus all other populations. In addition, a test for geographical structuring was performed.

### qRT-PCR analysis

Specific qRT-PCR primers were designed for 34 genes flanking 18 loci; in the case of loci 3, 7, 8, 9 and 17 unique primers for the most proximal genes could not be designed due to the surrounding repetitive sequence and thus the second most proximal gene was assayed. All primers were first verified using genomic DNA from insertion negative strains and amplified fragments verified by Sanger sequencing. Twelve genes were discarded due to unspecific primer binding identified through melting curve analysis or a lack of amplification from genomic DNA, or due to the presence of only one isolate in either the insertion positive or negative group, thus preventing statistical analysis of data. Total RNA was isolated using TRIzol® Reagent (Invitrogen) [43], reverse-transcribed into cDNA using the ImProm-II™ Reverse Transcription System (Promega), and qRT-PCR analysis was performed using a LightCycler 480 (Roche, Basel, Switzerland). Testing of the remaining 22 genes was done in duplicate in 20 μl reaction volumes using 1 μl of cDNA, 2 μl PCR Primer 10 × concentration and 10 μl of SYBR® Green Master Mix within the following cycling conditions: 5 minutes at 95°C (4 seconds at 95°C, 6 seconds at 55°C and 20 seconds at 72°C) × 45, and melting curve analysis (95°C for 1 second and 55°C for 1 second). Coronin (TVAG_021420), a known single copy gene previously characterized in different *T. vaginalis* isolates [18,19], was used as an endogenous control against which all gene expression normalizations were made. Amplification efficiencies were calculated for each primer set using cDNA from an insertion negative strain and relative mRNA levels were calculated for each gene in each isolate using the Pfaffl (2001) method [44]. Isolates were grouped based on their *Tvmar1* insertion on each locus. Significance of change in gene expression between these two groups was calculated by Welch Two Sample *t*-test using R version 2.13.1 (http://www.R-project.org). Linear regression analyses were carried out using R version 2.13.1.

## Abbreviations

AMOVA: Analysis of molecular variance; AUS: Australia; bp: Base pair; ITA: Italy; LST: Laboratory strains; Mb: Mega bases; MCMC: Markov chain Monte Carlo; MEX: Mexico; ORF: Open reading frame; PCA: Principal component analysis; PCR: Polymerase chain reaction; PNG: Papua New Guinea; qRT-PCR: Quantitative reverse transcription polymerase chain reaction; SAF: South Africa; SNP: Single nucleotide polymorphism; TE: Transposable element; TIR: Terminal inverted repeat; USA: United Sates of America.

## Competing interests

The authors declare that they have no competing interests.

## Authors’ contributions

MB designed the study, analyzed the data and drafted the manuscript. SW performed the gene expression experiments and participated in manuscript writing. VL carried out PCR insertion experiments and data analysis. JC participated in the design of the study and its coordination, and helped draft and finalize the manuscript. All authors read and approved the final manuscript.

## Supplementary Material

Additional file 1 **Alignment of the sequences of 19 *Tvmar1 *elements used in this study. (A)***Tvmar1* DNA alignment showing ITRs designated with a red box across the sequences. The transposase ORF is underlined. Indels that truncate the transposase ORF are indicated with arrowheads and non-synonymous mutations are designated with asterisks. ITR, inverted terminal repeat; ORF, open reading frame. Click here for file

Additional file 2 **Alignment of the sequences of 19 *Tvmar1 *elements used in this study. (B)** Protein alignment of *Tvmar1* transposase showing truncations and amino acid variation among the 19 elements. The aspartic acid residues that form the D, D34D catalytic motif are indicated with arrowheads. Click here for file

Additional file 3 **Table of 19 transposable elements used in this study.** The transposable element locus ID, primers used to amplify the loci and the flanking genes of the 19 transposable elements are shown. Click here for file

Additional file 4 **List of all *T. vaginalis *isolates used in this study.** Details of the geographical origin, year of isolation and reference if the isolate was previously described are provided. Click here for file

Additional file 5 **Table of insertion genotypes by locus and isolate.** The following labeling is used: 1, insertion positive; 0, insertion negative; NA, no data available; MIX, both PCR fragments present in the isolate. All MIX were treated as positive insertions in our data analysis. Click here for file

Additional file 6 **AMOVA in *T. vaginalis *global isolates for 19 *Tvmar1 *loci.** A result of no structuring was found to be significant among the groups (*P* <0.000001) with 9.96% of the explained variance, compared with 72.71% variance among individuals within populations. Contrasting the SAF group versus all other groups was significant (*P* <0.000001) and explained approximately 25% of the variance among groups, with the largest proportion of the variance among individuals within populations (58.70%), which did not show statistical support (*P* <0.05). % VAR, percentage of variation; AMOVA, analysis of molecular variance; SAF, South Africa; SS, sum of squares; VC, variance components. Click here for file

Additional file 7 **Table of qRT-PCR primers used in the study.** List of primers used to determine mRNA levels of genes flanking *Tvmar1* loci, and their description. Click here for file

Additional file 8 **Table of comparative parasite genetic types as determined by microsatellite and TE polymorphism data.** Parasite genetic type from microsatellite data in the table is based on microsatellite genotyping in Conrad *et al*. [19], while type from *Tvmar1* insertion data is based on this study. ND, not determined; TE, transposable element. Click here for file
