# Supplementary material for: The Tc1/mariner transposable element family shapes genetic variation and gene expression in the protist Trichomonas vaginalis
Source: Mob DNA. 2014 Apr 24;5:12. doi: 10.1186/1759-8753-5-12 (PMC4021607; doi:10.1186/1759-8753-5-12)
Supplement: Additional file 6 — AMOVA in T. vaginalis global isolates for 19 Tvmar1 loci. A result of no structuring was found to be significant among the groups (P <0.000001) with 9.96% of the explained variance, compared with 72.71% variance among individuals within populations. Contrasting the SAF group versus all other groups was significant (P <0.000001) and explained approximately 25% of the variance among groups, with the largest proportion of the variance among individuals within populations (58.70%), which did not show statistical support (P <0.05). % VAR, percentage of variation; AMOVA, analysis of molecular variance; SAF, South Africa; SS, sum of squares; VC, variance components. [file 1759-8753-5-12-S6.pdf]

Additional File 5. Analysis of molecular variance (AMOVA) in *T. vaginalis* global isolates for 19 *Tvmar1* loci.

Structure tested	SS	VC	%VAR	P
**One group: SAF, WUS, EUS, MEX, ITA, AUS, PNG, LAB**				
Among groups	40.11	0.14	9.96	0.00
Among individuals within populations	206.01	1.08	72.71	0.00
Within individuals	24.00	0.25	17.33	0.00
**Two groups: SAF *vs*. WUS, EUS, MEX, ITA, AUS, PNG, LAB**				
Among groups	19.78	0.46	24.99	0.00
Among populations within groups	20.32	0.04	2.32	0.00
Among individuals within populations	206.01	1.08	58.70	0.05
Within individuals	24.00	0.25	13.99	0.12
