# Supplementary material for: The Tc1/mariner transposable element family shapes genetic variation and gene expression in the protist Trichomonas vaginalis
Source: Mob DNA. 2014 Apr 24;5:12. doi: 10.1186/1759-8753-5-12 (PMC4021607; doi:10.1186/1759-8753-5-12)
Supplement: Additional file 2 — Alignment of the sequences of 19 Tvmar1 elements used in this study. (B) Protein alignment of Tvmar1 transposase showing truncations and amino acid variation among the 19 elements. The aspartic acid residues that form the D, D34D catalytic motif are indicated with arrowheads. [file 1759-8753-5-12-S2.pdf]

B.

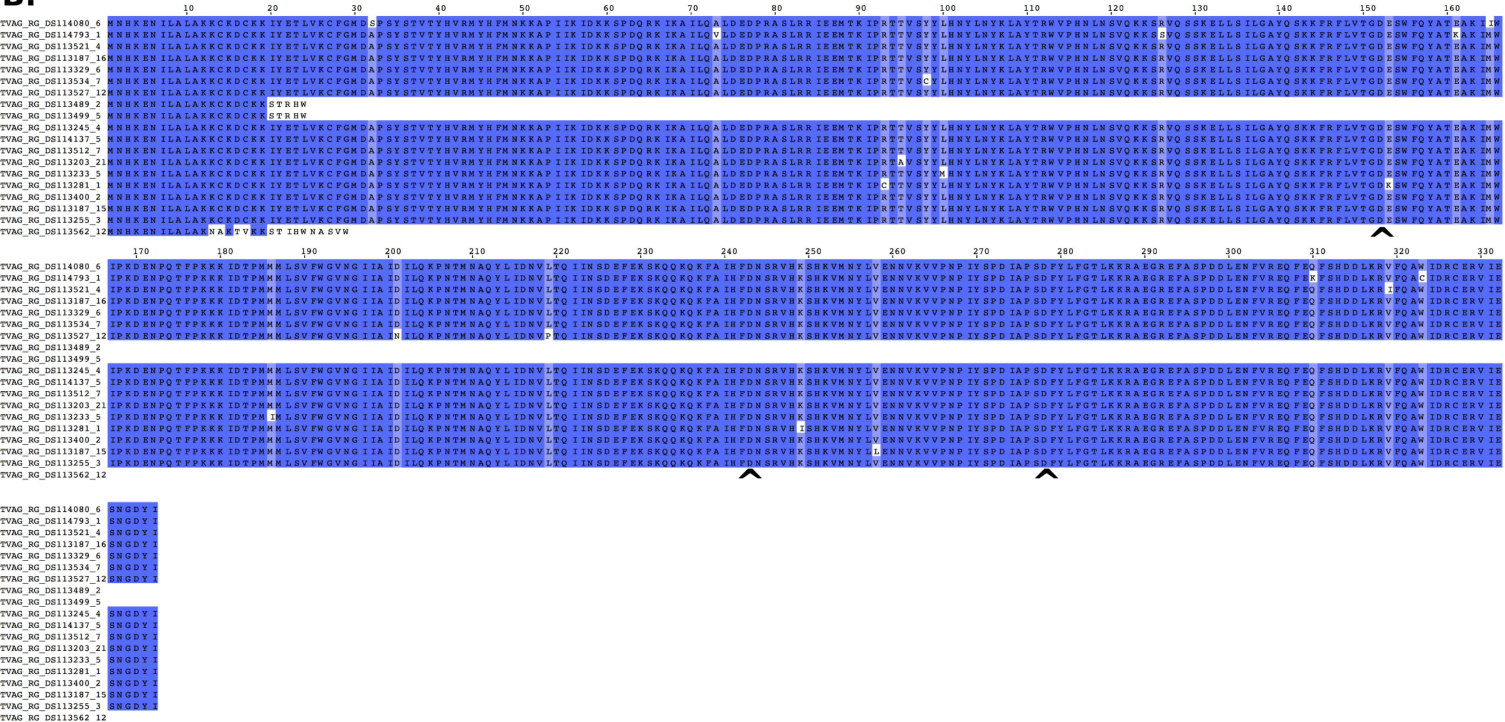
TVAG_RG_DS114080_6 MNHKENILALAKKCKDCKKIYETLVKCFGMDSPSYSTVTYHVRMYHFMNKKAPIIKIDKKSPDQRKIKAILQALDEDPRASLRRIEEMTKIPRTTVSYYLHNYLNYKLAYTRWVPHNLNSVQKKSRVQSSKELLSILGAYQSKKFRFLVTGDESWFQYATEAKIIW
TVAG_RG_DS114793_1 MNHKENILALAKKCKDCKKIYETLVKCFGMDAPSYSTVTYHVRMYHFMNKKAPIIKIDKKSPDQRKIKAILQVLDEDPRASLRRIEEMTKIPRTTVSYYLHNYLNYKLAYTRWVPHNLNSVQKKSSVQSSKELLSILGAYQSKKFRFLVTGDESWFQYATKAKIMW
TVAG_RG_DS113521_4 MNHKENILALAKKCKDCKKIYETLVKCFGMDAPSYSTVTYHVRMYHFMNKKAPIIKIDKKSPDQRKIKAILQALDEDPRASLRRIEEMTKIPRTTVSYYLHNYLNYKLAYTRWVPHNLNSVQKKSRVQSSKELLSILGAYQSKKFRFLVTGDESWFQYATEAKIMW
TVAG_RG_DS113187_16 MNHKENILALAKKCKDCKKIYETLVKCFGMDAPSYSTVTYHVRMYHFMNKKAPIIKIDKKSPDQRKIKAILQALDEDPRASLRRIEEMTKIPRTTVSYYLHNYLNYKLAYTRWVPHNLNSVQKKSRVQSSKELLSILGAYQSKKFRFLVTGDESWFQYATEAKIMW
TVAG_RG_DS113329_6 MNHKENILALAKKCKDCKKIYETLVKCFGMDAPSYSTVTYHVRMYHFMNKKAPIIKIDKKSPDQRKIKAILQALDEDPRASLRRIEEMTKIPRTTVSYYLHNYLNYKLAYTRWVPHNLNSVQKKSRVQSSKELLSILGAYQSKKFRFLVTGDESWFQYATEAKIMW
TVAG_RG_DS113534_7 MNHKENILALAKKCKDCKKIYETLVKCFGMDAPSYSTVTYHVRMYHFMNKKAPIIKIDKKSPDQRKIKAILQALDEDPRASLRRIEEMTKIPRTTVSCYLHNYLNYKLAYTRWVPHNLNSVQKKSRVQSSKELLSILGAYQSKKFRFLVTGDESWFQYATEAKIMW
TVAG_RG_DS113527_12 MNHKENILALAKKCKDCKKIYETLVKCFGMDAPSYSTVTYHVRMYHFMNKKAPIIKIDKKSPDQRKIKAILQALDEDPRASLRRIEEMTKIPRTTVSYYLHNYLNYKLAYTRWVPHNLNSVQKKSRVQSSKELLSILGAYQSKKFRFLVTGDESWFQYATEAKIMW
TVAG_RG_DS113489_2 MNHKENILALAKKCKDCKKSTRHW
TVAG_RG_DS113499_5 MNHKENILALAKKCKDCKKSTRHW
TVAG_RG_DS113245_4 MNHKENILALAKKCKDCKKIYETLVKCFGMDAPSYSTVTYHVRMYHFMNKKAPIIKIDKKSPDQRKIKAILQALDEDPRASLRRIEEMTKIPRTTVSYYLHNYLNYKLAYTRWVPHNLNSVQKKSRVQSSKELLSILGAYQSKKFRFLVTGDESWFQYATEAKIMW
TVAG_RG_DS114137_5 MNHKENILALAKKCKDCKKIYETLVKCFGMDAPSYSTVTYHVRMYHFMNKKAPIIKIDKKSPDQRKIKAILQALDEDPRASLRRIEEMTKIPRTTVSYYLHNYLNYKLAYTRWVPHNLNSVQKKSRVQSSKELLSILGAYQSKKFRFLVTGDESWFQYATEAKIMW
TVAG_RG_DS113512_7 MNHKENILALAKKCKDCKKIYETLVKCFGMDAPSYSTVTYHVRMYHFMNKKAPIIKIDKKSPDQRKIKAILQALDEDPRASLRRIEEMTKIPRTTVSYYLHNYLNYKLAYTRWVPHNLNSVQKKSRVQSSKELLSILGAYQSKKFRFLVTGDESWFQYATEAKIMW
TVAG_RG_DS113203_21 MNHKENILALAKKCKDCKKIYETLVKCFGMDAPSYSTVTYHVRMYHFMNKKAPIIKIDKKSPDQRKIKAILQALDEDPRASLRRIEEMTKIPRTAVSYYLHNYLNYKLAYTRWVPHNLNSVQKKSRVQSSKELLSILGAYQSKKFRFLVTGDESWFQYATEAKIMW
TVAG_RG_DS113233_5 MNHKENILALAKKCKDCKKIYETLVKCFGMDAPSYSTVTYHVRMYHFMNKKAPIIKIDKKSPDQRKIKAILQALDEDPRASLRRIEEMTKIPRTTVSYYMHNYLNYKLAYTRWVPHNLNSVQKKSRVQSSKELLSILGAYQSKKFRFLVTGDESWFQYATEAKIMW
TVAG_RG_DS113281_1 MNHKENILALAKKCKDCKKIYETLVKCFGMDAPSYSTVTYHVRMYHFMNKKAPIIKIDKKSPDQRKIKAILQALDEDPRASLRRIEEMTKIPCTTVSYYLHNYLNYKLAYTRWVPHNLNSVQKKSRVQSSKELLSILGAYQSKKFRFLVTGDKSWFQYATEAKIMW
TVAG_RG_DS113400_2 MNHKENILALAKKCKDCKKIYETLVKCFGMDAPSYSTVTYHVRMYHFMNKKAPIIKIDKKSPDQRKIKAILQALDEDPRASLRRIEEMTKIPRTTVSYYLHNYLNYKLAYTRWVPHNLNSVQKKSRVQSSKELLSILGAYQSKKFRFLVTGDESWFQYATEAKIMW
TVAG_RG_DS113187_15 MNHKENILALAKKCKDCKKIYETLVKCFGMDAPSYSTVTYHVRMYHFMNKKAPIIKIDKKSPDQRKIKAILQALDEDPRASLRRIEEMTKIPRTTVSYYLHNYLNYKLAYTRWVPHNLNSVQKKSRVQSSKELLSILGAYQSKKFRFLVTGDESWFQYATEAKIMW
TVAG_RG_DS113255_3 MNHKENILALAKKCKDCKKIYETLVKCFGMDAPSYSTVTYHVRMYHFMNKKAPIIKIDKKSPDQRKIKAILQALDEDPRASLRRIEEMTKIPRTTVSYYLHNYLNYKLAYTRWVPHNLNSVQKKSRVQSSKELLSILGAYQSKKFRFLVTGDESWFQYATEAKIMW
TVAG_RG_DS113562_12 MNHKENILALAKNAKTVKKSTIHWNASVW
TVAG_RG_DS114080_6 IPKDENPQTFPKKKIDTPMMMLSVFWGVNGIIAIDILQKPNTMNAQYLIDNVLTQIINSDEFEKSKQQKQKFAIHFDNSRVHKSHKVMNYLVENNVKVVPNPIYSPDIAPSDFYLFGTLKKRAEGREFASPDDLENFVREQFEQFSHDDLKRVFQAWIDRCERVIE
TVAG_RG_DS114793_1 IPKDENPQTFPKKKIDTPMMMLSVFWGVNGIIAIDILQKPNTMNAQYLIDNVLTQIINSDEFEKSKQQKQKFAIHFDNSRVHKSHKVMNYLVENNVKVVPNPIYSPDIAPSDFYLFGTLKKRAEGREFASPDDLENFVREQFEKFSHDDLKRVFQACIDRCERVIE
TVAG_RG_DS113521_4 IPKDENPQTFPKKKIDTPMMMLSVFWGVNGIIAIDILQKPNTMNAQYLIDNVLTQIINSDEFEKSKQQKQKFAIHFDNSRVHKSHKVMNYLVENNVKVVPNPIYSPDIAPSDFYLFGTLKKRAEGREFASPDDLENFVREQFEQFSHDDLKRIFQAWIDRCERVIE
TVAG_RG_DS113187_16 IPKDENPQTFPKKKIDTPMMMLSVFWGVNGIIAIDILQKPNTMNAQYLIDNVLTQIINSDEFEKSKQQKQKFAIHFDNSRVHKSHKVMNYLVENNVKVVPNPIYSPDIAPSDFYLFGTLKKRAEGREFASPDDLENFVREQFEQFSHDDLKRVFQAWIDRCERVIE
TVAG_RG_DS113329_6 IPKDENPQTFPKKKIDTPMMMLSVFWGVNGIIAIDILQKPNTMNAQYLIDNVLTQIINSDEFEKSKQQKQKFAIHFDNSRVHKSHKVMNYLVENNVKVVPNPIYSPDIAPSDFYLFGTLKKRAEGREFASPDDLENFVREQFEQFSHDDLKRVFQAWIDRCERVIE
TVAG_RG_DS113534_7 IPKDENPQTFPKKKIDTPMMMLSVFWGVNGIIAIDILQKPNTMNAQYLIDNVLTQIINSDEFEKSKQQKQKFAIHFDNSRVHKSHKVMNYLVENNVKVVPNPIYSPDIAPSDFYLFGTLKKRAEGREFASPDDLENFVREQFEQFSHDDLKRVFQAWIDRCERVIE
TVAG_RG_DS113527_12 IPKDENPQTFPKKKIDTPMMMLSVFWGVNGIIAINILQKPNTMNAQYLIDNVPTQIINSDEFEKSKQQKQKFAIHFDNSRVHKSHKVMNYLVENNVKVVPNPIYSPDIAPSDFYLFGTLKKRAEGREFASPDDLENFVREQFEQFSHDDLKRVFQAWIDRCERVIE
TVAG_RG_DS113489_2
TVAG_RG_DS113499_5
TVAG_RG_DS113245_4 IPKDENPQTFPKKKIDTPMMMLSVFWGVNGIIAIDILQKPNTMNAQYLIDNVLTQIINSDEFEKSKQQKQKFAIHFDNSRVHKSHKVMNYLVENNVKVVPNPIYSPDIAPSDFYLFGTLKKRAEGREFASPDDLENFVREQFEQFSHDDLKRVFQAWIDRCERVIE
TVAG_RG_DS114137_5 IPKDENPQTFPKKKIDTPMMMLSVFWGVNGIIAIDILQKPNTMNAQYLIDNVLTQIINSDEFEKSKQQKQKFAIHFDNSRVHKSHKVMNYLVENNVKVVPNPIYSPDIAPSDFYLFGTLKKRAEGREFASPDDLENFVREQFEQFSHDDLKRVFQAWIDRCERVIE
TVAG_RG_DS113512_7 IPKDENPQTFPKKKIDTPMMMLSVFWGVNGIIAIDILQKPNTMNAQYLIDNVLTQIINSDEFEKSKQQKQKFAIHFDNSRVHKSHKVMNYLVENNVKVVPNPIYSPDIAPSDFYLFGTLKKRAEGREFASPDDLENFVREQFEQFSHDDLKRVFQAWIDRCERVIE
TVAG_RG_DS113203_21 IPKDENPQTFPKKKIDTPMMMLSVFWGVNGIIAIDILQKPNTMNAQYLIDNVLTQIINSDEFEKSKQQKQKFAIHFDNSRVHKSHKVMNYLVENNVKVVPNPIYSPDIAPSDFYLFGTLKKRAEGREFASPDDLENFVREQFEQFSHDDLKRVFQAWIDRCERVIE
TVAG_RG_DS113233_5 IPKDENPQTFPKKKIDTPMIMLSVFWGVNGIIAIDILQKPNTMNAQYLIDNVLTQIINSDEFEKSKQQKQKFAIHFDNSRVHKSHKVMNYLVENNVKVVPNPIYSPDIAPSDFYLFGTLKKRAEGREFASPDDLENFVREQFEQFSHDDLKRVFQAWIDRCERVIE
TVAG_RG_DS113281_1 IPKDENPQTFPKKKIDTPMMMLSVFWGVNGIIAIDILQKPNTMNAQYLIDNVLTQIINSDEFEKSKQQKQKFAIHFDNSRVHISHKVMNYLVENNVKVVPNPIYSPDIAPSDFYLFGTLKKRAEGREFASPDDLENFVREQFEQFSHDDLKRVFQAWIDRCERVIE
TVAG_RG_DS113400_2 IPKDENPQTFPKKKIDTPMMMLSVFWGVNGIIAIDILQKPNTMNAQYLIDNVLTQIINSDEFEKSKQQKQKFAIHFDNSRVHKSHKVMNYLVENNVKVVPNPIYSPDIAPSDFYLFGTLKKRAEGREFASPDDLENFVREQFEQFSHDDLKRVFQAWIDRCERVIE
TVAG_RG_DS113187_15 IPKDENPQTFPKKKIDTPMMMLSVFWGVNGIIAIDILQKPNTMNAQYLIDNVLTQIINSDEFEKSKQQKQKFAIHFDNSRVHKSHKVMNYLLENNVKVVPNPIYSPDIAPSDFYLFGTLKKRAEGREFASPDDLENFVREQFEQFSHDDLKRVFQAWIDRCERVIE
TVAG_RG_DS113255_3 IPKDENPQTFPKKKIDTPMMMLSVFWGVNGIIAIDILQKPNTMNAQYLIDNVLTQIINSDEFEKSKQQKQKFAIHFDNSRVHKSHKVMNYLVENNVKVVPNPIYSPDIAPSDFYLFGTLKKRAEGREFASPDDLENFVREQFEQFSHDDLKRVFQAWIDRCERVIE
TVAG_RG_DS113562_12
TVAG_RG_DS114080_6 SNGDYI
TVAG_RG_DS114793_1 SNGDYI
TVAG_RG_DS113521_4 SNGDYI
TVAG_RG_DS113187_16 SNGDYI
TVAG_RG_DS113329_6 SNGDYI
TVAG_RG_DS113534_7 SNGDYI
TVAG_RG_DS113527_12 SNGDYI
TVAG_RG_DS113489_2
TVAG_RG_DS113499_5
TVAG_RG_DS113245_4 SNGDYI
TVAG_RG_DS114137_5 SNGDYI
TVAG_RG_DS113512_7 SNGDYI
TVAG_RG_DS113203_21 SNGDYI
TVAG_RG_DS113233_5 SNGDYI
TVAG_RG_DS113281_1 SNGDYI
TVAG_RG_DS113400_2 SNGDYI
TVAG_RG_DS113187_15 SNGDYI
TVAG_RG_DS113255_3 SNGDYI
TVAG_RG_DS113562_12
